# How many neurons can we see with current spike sorting algorithms?

**DOI:** 10.1016/j.jneumeth.2012.07.010

**Published:** 2012-10-15

**Authors:** Carlos Pedreira, Juan Martinez, Matias J. Ison, Rodrigo Quian Quiroga

**Affiliations:** Department of Engineering, University of Leicester, UK

**Keywords:** Spike-sorting, Sparse neurons, Extracellular recordings

## Abstract

Recent studies highlighted the disagreement between the typical number of neurons observed with extracellular recordings and the ones to be expected based on anatomical and physiological considerations. This disagreement has been mainly attributed to the presence of sparsely firing neurons. However, it is also possible that this is due to limitations of the spike sorting algorithms used to process the data. To address this issue, we used realistic simulations of extracellular recordings and found a relatively poor spike sorting performance for simulations containing a large number of neurons. In fact, the number of correctly identified neurons for single-channel recordings showed an asymptotic behavior saturating at about 8–10 units, when up to 20 units were present in the data. This performance was significantly poorer for neurons with low firing rates, as these units were twice more likely to be missed than the ones with high firing rates in simulations containing many neurons. These results uncover one of the main reasons for the relatively low number of neurons found in extracellular recording and also stress the importance of further developments of spike sorting algorithms.

## Highlights

► Spike sorting algorithms are limited in the number of single units they can detect. ► The maximum number of correctly identified neurons stands between 8 and 10. ► Sparse neurons are strongly affected by this limitation. ► Further development of algorithms is needed to address sparse neurons detection.

## 1 Introduction

Complex brain processes are encoded by the activity of relatively large neural networks and, consequently, the study of these processes can benefit from the simultaneous recording and analysis of the firing patterns from large populations of neurons (Douglas and Martin, 1991; Brown et al., 2004; Harris et al., 2003; Buzsaki, 2004; Quian Quiroga and Panzeri, 2009). In addition, applications using Brain Machine Interfaces and neural prostheses could significantly profit from the possibility of recording large numbers of neurons, as this allows the decoding of more complex and precise motor actions (Donoghue, 2002; Carmena et al., 2003; Chapin, 2004; Musallam et al., 2004; Velliste et al., 2008; Nicolelis and Lebedev, 2009). The development of multiple electrode recording probes (Maynard et al., 1997; Blanche et al., 2005; Keefer et al., 2008) and spike sorting algorithms (Letelier and Weber, 2000; Pouzat et al., 2002; Quian Quiroga et al., 2004; Zhang et al., 2004; Rutishauser et al., 2006; Vargas-Irwin and Donoghue, 2007) have provided an increasing number of identified neurons from multiple-site recordings, reaching values of dozens or even hundreds of simultaneously recorded neurons over long periods of time (Carmena et al., 2003; Buzsaki, 2004; Blanche et al., 2005; Jackson and Fetz, 2007; Tolias et al., 2007). In the case of single channel recordings, improvements of spike sorting algorithms have allowed identifying up to half a dozen neurons, as it is the case with data from the human medial temporal lobe (Quian Quiroga et al., 2005, 2007).

In spite of these advances, based on the effective radius seen by an electrode and the density of neurons, it has been estimated that the number of identified neurons per channel should be one or two orders of magnitude higher (Henze et al., 2000; Buzsaki, 2004). Different factors have been proposed to account for the relatively low number of identified neurons. In particular, it was suggested that this could be due to tissue damage caused by the insertion of the electrodes in the recording area (Claverol-Tinture and Nadasdy, 2004), or the electrical insulation caused by the substrate of the probe (Moffitt and McIntyre, 2005). Another possible reason for this mismatch is the presence of sparsely firing neurons, which are unlikely to be detected because they are silent most of the time (Buzsaki, 2004; Shoham et al., 2006). Nonetheless, even the combination of these effects can hardly account for the large gap between the expected number of units and the ones obtained in real data. In this study we evaluate yet another possibility, namely, that the relatively low number of detected neurons could be due to limitations of the spike sorting algorithms.

Let us illustrate this issue with a simple calculation. Approximating the neuron by a dipole source, following Coulomb's law the amplitude decays as *v* ∼ *1*/*r*^*2*^, as a function of the distance. We can also consider that spikes of about 60 μV are produced by neurons at a distance of about 50 μm – the maximum distance (and minimum amplitude) at which single units are identified (Buzsaki, 2004). Taking a density of 300,000 neurons/mm^3^ (as in the rat hippocampus; Henze et al., 2000), it immediately follows that there should be more than 30 neurons firing spikes with an amplitude between 60 and 70 μV, at the threshold of single unit detection, and about 10 neurons firing (relatively large) spikes, within 100 and 110 μV. So, within a small amplitude range of 10 μV there are several neurons whose spikes could hardly be separated by their amplitudes (*i.e.* using a spike sorter based on amplitude discrimination) given the background noise. It is also likely that more sophisticated spike sorters that extract features of the spike shapes (beyond their amplitude) will also miss at least some of these neurons, as we report below.

With some notable exceptions (see Harris et al., 2000 and Wehr et al., 1999 for examples of simultaneous extra- and intra-cellular recordings), a major limitation in the evaluation of spike sorting algorithms with real data is that there is no ‘ground truth’ to univocally obtain a measure of an algorithm's performance. Consequently, the quantification of spike sorting results requires the use of synthetic datasets, which also offer the possibility of controlling parameters such as the signal to noise ratio, the firing rate of the units and the characteristics of the different spike shapes, thus allowing a reliable measure of the algorithm performance under different recording conditions. In line, synthetic datasets were used in several previous works (Letelier and Weber, 2000; Quian Quiroga et al., 2004; Zhang et al., 2004; Vargas-Irwin and Donoghue, 2007). However, these spike sorting outcomes were tested with simulations containing only a few single-units. Therefore, it remains unknown how these algorithms would perform on datasets with larger number of neurons, as it can be expected from the considerations discussed above. To address this issue, in this study we systematically evaluated the spike sorting performance using simulations of extracellular recordings with increasing number of neurons, also focusing on the detection of sparsely firing neurons.

## 2 Materials and methods

**Fig. 1:**
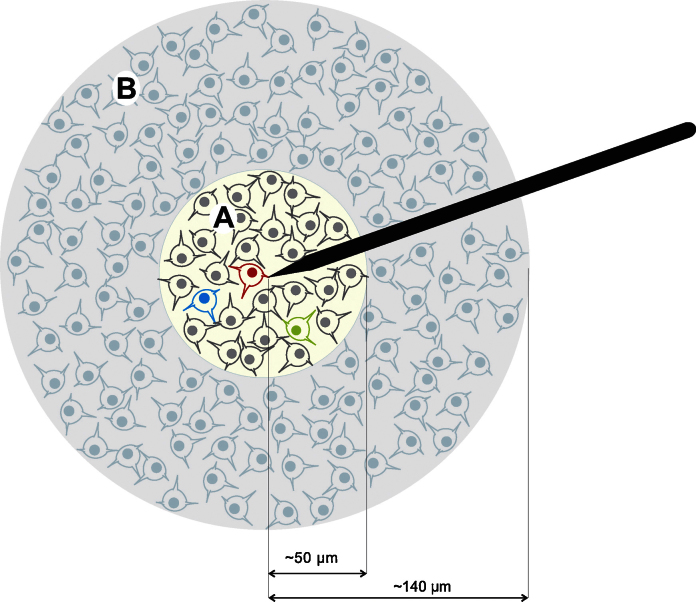
Extracellular recordings. Spikes in the area close to the electrode tip (region A) are considerably higher than the background noise, and are therefore easily detected and sorted. The neurons in red, green and cyan represent 3 active neurons recorded by the electrode and sorted by the algorithm. The neurons in gray represent those that fire very sparsely and are not detected. Neurons in the light gray area (B) produce spikes that are larger than the background activity, but not large enough to be individually sorted. The spikes fired by these neurons are usually grouped together in a multiunit cluster. Neurons outside this area contribute to the background noise.

Neurons contributing to the extracellular recording can be seen as generators of electric signals that are captured by the electrode placed in the brain tissue. When activated, each of these generators produce action potentials (or spikes) with a particular amplitude and shape (Gold et al., 2006). A simplified scheme of the recording with an electrode implanted in neural tissue is shown in Fig. 1. In the area surrounding the tip of the electrode (region A, with white background) the magnitude of the spikes is considerably higher than the background noise and it is therefore possible to detect and identify the firing of the neurons with high accuracy. The neurons in red, green and cyan represent active neurons in this area – up to 50 μm from the electrode tip (Buzsaki, 2004) – recorded by the electrode and sorted according to their shapes. The remaining neurons in this area (in gray) represent neurons whose activity is not detected due to their very low firing rate (see Shoham et al., 2006). The area in light gray (region B) contains neurons close enough to the tip of the electrode to produce spikes larger than the overall noise level, but too small to be individually sorted. The spikes fired by these neurons are usually grouped together and are considered to be a ‘multiunit cluster’. Neurons further away from the electrode tip – outside area B, *i.e.* more than 140 μm from the electrode tip (Buzsaki, 2004) – produce spikes that are too small to be detected and they only contribute to the background noise.

To evaluate the spike sorting performance with different numbers of neurons, we created a total of 95 simulations of 10 min of extracellular recordings. Each of these simulations contained background noise, multiunit activity and between 2 and 20 neurons (5 simulations for each case). Following previous studies from our group (Quian Quiroga et al., 2004; Martinez et al., 2009), the noise, multiunit and single unit activities were generated using a database of 594 different average spikes compiled from recordings of monkey basal ganglia and neocortex, as detailed below. In the simulated data, the spike times and labels of the multiunit and the single unit spikes were stored for subsequent analyses.

The data was first generated with a sample rate of 96,000 Hz and it was then decimated by a factor of 4, thus giving a sampling rate of 24,000 Hz. This replicated the fact that in real recording conditions spikes occur at continuous time points, thus introducing misalignments and making spike sorting more challenging.

### 2.1 Simulation of the background noise

For each simulation, the first step was to generate the background noise by modeling the overall contribution of distant neurons (neurons at a distance larger than 140 μm from the electrode tip; area outside the circle shown in Fig. 1). To do this, we superimposed at random times a large number of spikes selected randomly from the average spike shapes in the database. As in previous works (Quian Quiroga et al., 2004; Martinez et al., 2009), the total number of spikes used to build the noise was half the number of samples during the generation of the signal, prior to the downsampling process, *i.e.* each second of simulation was generated by the superimposition of 48,000 spikes. The amplitude of each of these spikes was scaled by a value randomly selected from a normal distribution (*μ* = 1, *σ* = 0.2). After superposition of all the spikes, the mean of the resulting signal was then subtracted and scaled to a standard deviation of 0.1.

### 2.2 Simulation of multiunit activity

The simulated signals also included multiunit activity, generated by the spikes fired by neurons in region B of Fig. 1. Although the aim of our study was to analyze the clustering performance for the single units, the multiunit activity was introduced in order to replicate a more realistic scenario where the presence of large multiunit clusters increased the complexity of the sorting process.

To simulate the multiunit activity we added the contribution of 20 different spike shapes randomly selected from the database to the background noise. The amplitude of the spikes used to construct the multiunit activity was fixed to 0.5, a value close to the detection threshold (see below). As typically found in the experimental data, the firing rate of the multiunit was set to 5 Hz, *i.e.*, each of the 20 neurons generating the multiunit followed an independent Poisson distribution with a mean firing rate of 0.25 Hz.

### 2.3 Simulation of single unit activity

Single unit activity is given by the firing of neurons close-by the electrode tip (region A in Fig. 1). The time of firing of the single units was modeled following a Poisson distribution with a mean firing rate randomly selected between 0.1 and 2 Hz (uniform probability). The amplitude of each unit was randomly selected from a normal distribution (*μ* = 1.1, *σ* = 0.5), capped to values within the range 0.9–2 to resemble the amplitude distribution (referred to the noise level) found in real data (Quian Quiroga et al., 2007). For the sake of clarity we did not to include overlapping spikes in our simulations (*i.e.* spikes had to be more than 3 ms apart from each other). It is likely that the limitation of spike sorting algorithms described in our results will be even larger when considering overlapping spikes.

### 2.4 Performance evaluation

Three expert operators performed an independent (and blind) spike sorting of the 95 simulations – each with 2–20 neurons – using Wave_clus (Quian Quiroga et al., 2004). This software offers a combination of properties specially suited for our study: (i) the detection threshold is based on the median of the absolute value of the signal, thus offering a robust estimation of the background noise and reducing the dependence on the amount (and amplitude) of the spikes present in the signal; (ii) the number of clusters obtained is determined automatically; (iii) the algorithm does not make *a priori* assumptions about the shape of the clusters; and (iv) it is able to identify clusters with very different sizes, which is well-suited for clustering sparsely firing neurons. The sorting process for each simulation was performed automatically using the default parameters of Wave_clus (Quian Quiroga et al., 2004). When the automatic clustering was not satisfactory, the operators optimized the results using the Wave_clus GUI (by changing the clustering temperature, merging or rejecting clusters).

For each spike sorting outcome, we quantified the number of correctly and incorrectly identified neurons using the following criteria. *Hits* referred to correctly identified clusters fulfilling two conditions: (1) at least 50% of the spikes corresponded to the same neuron; and (2) the number of detected spikes were, at least, 50% of the number of generated spikes for this particular neuron. As shown in Fig. S1, other choices gave similar results. The number of *misses* was quantified as the total number of generated neurons minus the number of hits. In addition, the clusters identified by the user that did not fulfill condition (1) or (2) were considered to be *false positives*, and they corresponded to spurious subdivisions of the units present in the simulation.

Given the previously reported problems in separating clusters with relatively large size differences (Ott et al., 2005), we also divided our units into low-firing and high-firing neurons for additional analysis. We defined low-firing neurons as those with a firing rate below 0.5 Hz (less than 300 spikes in the 10 min of simulated data) and high-firing neurons as the rest, and evaluated the performance for both populations separately. Due to the random mean firing rate assigned to each unit, for each on of the simulations with a certain number of units we obtained different number of low-firing and high-firing neurons. Therefore, to compare both populations we defined the performance ratio as the number of hits with high- (low-) firing neurons, divided by the total number of high- (low-) firing neurons present in the simulation. Ratios for misses and false positives were defined in a similar way.

### 2.5 Statistical analysis

The spike sorting performance for the low- and high-firing neurons was compared using a two-way ANOVA (Test 1). The two independent variables were the number of neurons in each simulation (from 2 to 20) and the neuron type (*i.e.* low-firing or high-firing). The repeated measures were the corresponding performance ratios (hits, misses or false positives).

To ensure that there was no statistical difference between results obtained by the three experts we tested for inter-rater reliability using a Fleiss’ kappa coefficient for hits-false positives results as well as for the hits for the low-firing and high-firing units (Test 2).

## 3 Results

### 3.1 Single case examples

**Fig. 2:**
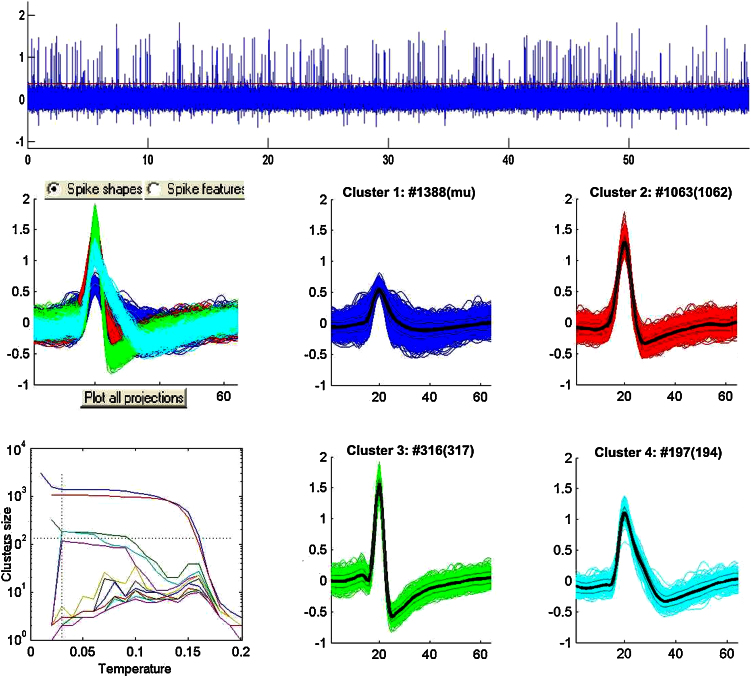
Example of spike sorting. The top plot shows 60 s of simulated data. The bottom plots show the superposition of all the spike clusters (left) and each of the sorted clusters, corresponding to one multiunit (cluster 1) and 3 single units (clusters 2, 3 and 4). The number on top of each plot indicates the number of spikes generated (in brackets) and detected by the user.

An example of the simulated data and the spike sorting performed by one of the experts is shown in Fig. 2. The top plot shows 60 s of raw signal and the bottom plots show the (correctly) sorted units: 3 single units (clusters 2, 3 and 4) and 1 multiunit (cluster 1). The simulation contained background noise, a multiunit activity cluster 2916 spikes, and 3 single units (classes 1, 2 and 3) with amplitudes 1.38, 1.69 and 1.22 and firing rates 1.91, 0.52 and 0.35 Hz, which gave 1063, 317 and 194 spikes, respectively. For the multiunit, only 1338 out of 2916 spikes were correctly identified by the user. The multiunit cluster contained 50 false detections and the remaining 1522 spikes did not cross the detection threshold. The detection and sorting performance of single units was, as expected, much more accurate than the one of multiunit. For cluster 2 the operator detected 1062 of the 1063 spikes generated for this unit plus a single spike from another unit. For cluster 3 the operator detected 316 of the 317 generated spikes. For cluster 4 the operator detected all the 194 spikes, plus 3 extra spikes from other units.

**Fig. 3:**
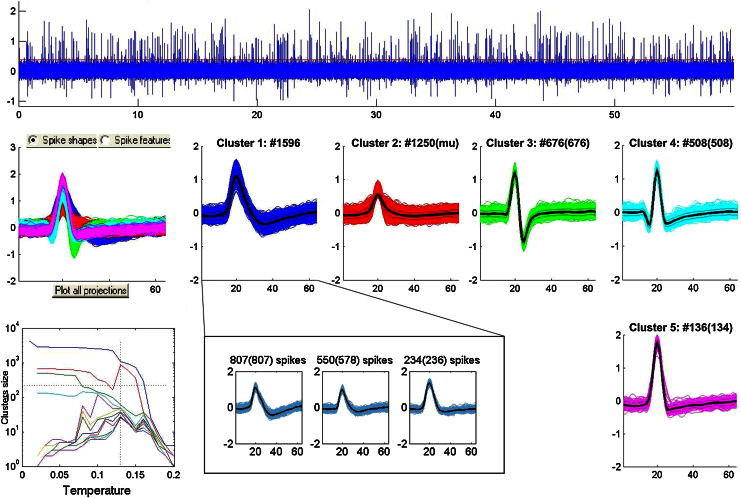
Spike sorting with a large number of units. For this simulation, the operator incorrectly identified one multiunit (cluster 2) and 4 single units (clusters 1, 3, 4 and 5). However, cluster 1 was formed by 3 different neurons (inset).

Fig. 3 shows a more complex simulation with 1 multiunit and 6 single units. In this case, the simulation was originally generated with a multiunit containing a total of 2870 spikes and the single units (classes 1–6) had amplitudes 1.95, 1.35, 1.19, 1.08, 1.57 and 1.3, and firing rates 0.24, 0.85, 1.54, 1.02, 0.43 and 1.19 Hz, respectively. The operator clustered a multiunit (cluster 2) and 4 single units (clusters 1, 3, 4 and 5). For clusters 3 and 4 the operator correctly identified 676 and 508 spikes, respectively. For cluster 5 the operator detected all the 134 spikes and 2 more spikes from another neuron. In contrast, cluster 1 was incorrectly identified as a single unit. In fact, this cluster contained the activity of 3 different neurons (shown in the inset of the figure) with quite overlapping shapes that appeared as a single cluster to the operator. This error was partially due to the presence of other neurons in the simulation. The clustering of only these 3 spike shapes indeed gave the correct result (see Fig. S2).

### 3.2 User's performance

Three different operators blindly sorted the 95 simulations generated for this study (see Section 2). Table 1 shows the number of hits for all simulations, for each operator separately. Despite some individual differences, all users had a similar overall performance, significantly different from coincidental agreement (Test 2 for hits minus false positives, kappa = 0.33, *z* = 16.67, *p* < 10^−5^), reaching a similar asymptotic average number of hits in their sorting results. This suggests that the results obtained for the different number of neurons were related to the inherent characteristics of the spike sorting process and were not significantly influenced by the subjectivity and potential biases of each operator. Similar results were found when dividing the results by firing rates, with significant coincidences for both high-firing and low-firing units (Test 2, high-firing hits: kappa = 0.55, *z* = 25.87, *p* < 10^−5^ low-firing hits: kappa = 0.56, *z* = 15.09, *p* < 10^−5^). Table 2 presents the number of false positives. Altogether, the number of false positives was relatively low, thus indicating that overclustering (*i.e.* splitting the activity of a single source – simulated neuron – into 2 or more clusters) was rare.

### 3.3 Number of identified neurons

**Fig. 4:**
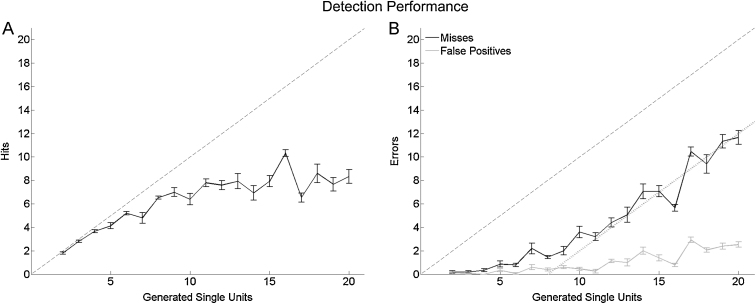
Spike sorting performance with increasing number of neurons. The mean detection performance is shown as a function of the number of single units present in the simulation. (A) Average number of hits. (B) Average number of misses (black) and false positives (dashed gray). For the misses, the dotted gray line in B shows the linear function *y* = *x* − 8; *i.e.* a line parallel to the diagonal but displaced 8, the maximum number of neurons detected. Bars denote s.e.m.

Fig. 4a shows the average number of hits as a function of the number of generated neurons. For simulations with a few neurons the number of detected clusters was nearly perfect (the ideal performance marked by the dashed line). For simulations with more neurons the performance decayed compared to the ideal performance, reaching an asymptotic value at about 8–10 correctly identified neurons. Fig. 4b displays the number of misses and false positives for the different number of generated neurons. The curve with the misses shows a complementary behavior to the one of the number of hits. It is negligible for low number of units and it rises as the number of generated neurons increase. In fact, the dotted line in Fig. 4b (*y* = *x* − 8) fits closely the number of misses in agreement with the asymptotic behavior showed by the hits. Meanwhile, the number of false positives also increased with the number of units present in the simulation, but it remained below 3. These results indicate that the decrease in detection performance was due to the grouping two or more neurons into single clusters, as exemplified in Fig. 3.

### 3.4 Sparse neurons

**Fig. 5:**
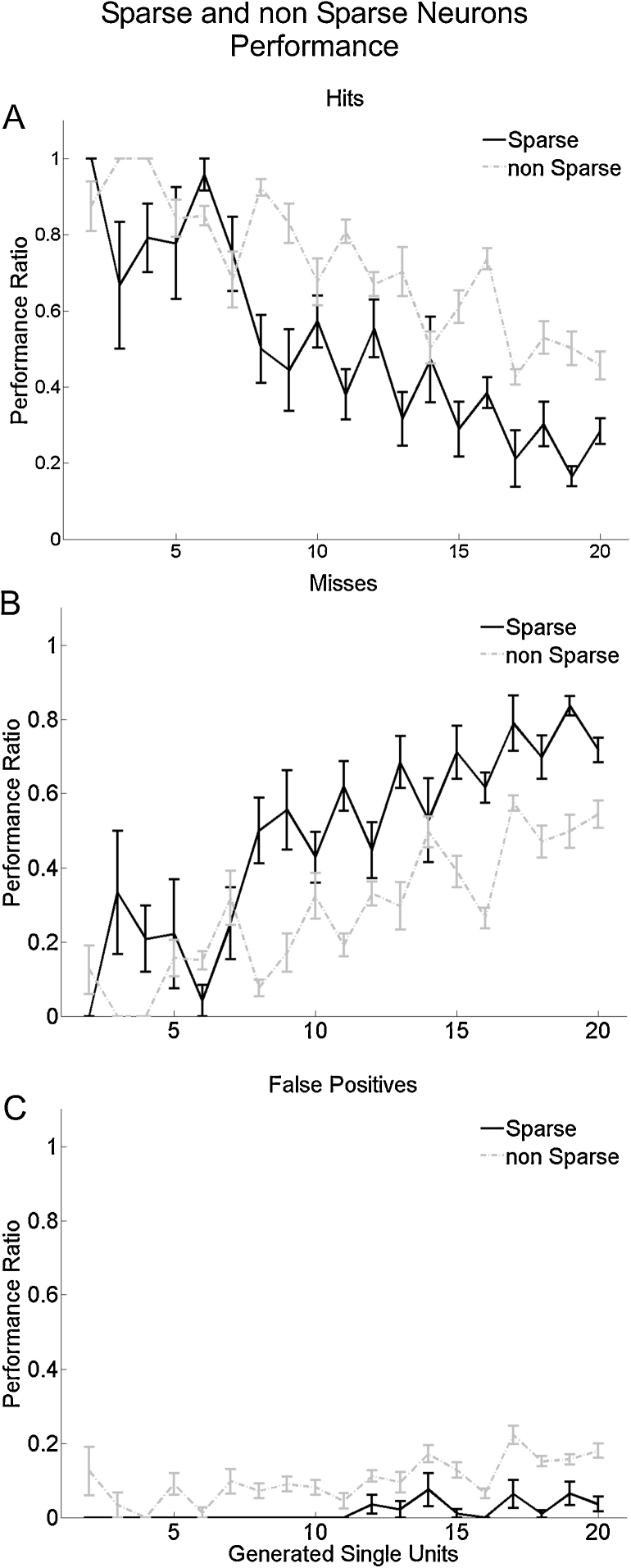
Hits and false positive ratios for the sparse and non-sparse neurons. (A) Number of hits for sparse (dark gray) and non-sparse neurons (light gray), Number of misses (B) and false positives (C) for the same groups. Bars denote s.e.m.

To quantify the spike sorting performance for different types of neurons, we divided our data into low- and high-firing neurons (see Section 2). Fig. 5a shows the hit ratio for both types of neurons and Fig. 5b and c the number of misses and false positives respectively. It can be seen that the performance for high-firing neurons was better than for the low-firing ones. For the cases with a large number of neurons, the ratio of misses for the low-firing neurons was around 80%, while for the high-firing neurons it reached values around 50%. These differences were statistically significant (*F*(1,511) = 104.08, *p* < 10^−15^, *F*(1,511) = 129.76, *p* < 10^−15^, Test 1, for hits and false positives, respectively). These results show the tendency of sparse neurons to be masked by multiunit activity or by cells with high firing rates.

## 4 Discussion

Recent studies have drawn attention to the apparent discrepancy between the number of neurons one expects to see following anatomical and physiological considerations – in the order of few hundred – and the number of neurons typically detected in experimental conditions with single electrodes – up to 6 – (Buzsaki, 2004; Shoham et al., 2006). This difference has been commonly attributed to the sparseness of neural firing (Henze et al., 2000; Olshausen and Field, 2004; Shoham et al., 2006; Fujisawa et al., 2008) and the damage of the neurological tissue around the electrode (Claverol-Tinture and Nadasdy, 2004). In addition to these factors, here we showed that limitations of spike sorting algorithms also contribute to the relative low number of detected neurons.

### 4.1 Limitations of spike sorting algorithms

Previous studies have analyzed and characterized the performance of several spike sorting methods (Letelier and Weber, 2000; Pouzat et al., 2002; Quian Quiroga et al., 2004; Zhang et al., 2004; Rutishauser et al., 2006; Vargas-Irwin and Donoghue, 2007). All these studies have, however, used relatively few neurons, thus not exploring the limits of spike sorting algorithms. Here we showed that the number of identified neurons reached a maximum of about 8–10, even when up to 20 neurons were present in the signal. Interestingly, there were relatively few false positives. Hence, the sorting mistakes were mainly produced by grouping 2 or more neurons into a single cluster, an effect also observed in previous studies but with a lower number of units (Blanche et al., 2005; Kwon et al., 2012). In addition, to show that this limitation of spike sorting is not exclusive of our own algorithm (Wave_clus), we analyzed the same set of simulations using a completely different and well-known algorithm, KlustaKwik (Harris et al., 2000), and obtained very similar results to the ones presented here.

### 4.2 Sparsely firing neurons

Our simulations included single units with a wide range of firing rates, as reported in experimental recordings (Musallam et al., 2004; Quian Quiroga et al., 2005; Fujisawa et al., 2008). The sorting results for low- and high-firing neurons were significantly different and showed a better performance for the latter (see Fig. 5), despite the reported ability of the used algorithm to identify sparsely firing neurons in real recordings (Quian Quiroga et al., 2005, 2007, 2008). This is due to the fact that the activity of sparsely firing units is typically masked by the activity of neurons with higher firing rates recorded from the same channel. In other words, sparsely firing neurons show clusters with relatively few spikes which are typically merged with larger close-by clusters. A possible improvement could be to implement an iterative procedure to sort the remaining spikes after eliminating already identified (and large) clusters (Ott et al., 2005). However, the success of this approach is not guaranteed because the eliminated clusters could already include the activity of the sparsely firing neurons.

It has been reported that firing rates can be even lower (<0.05 spikes/s) than the ones we used in our simulations (Olshausen and Field, 2004; Shoham et al., 2006). Given the problem in detecting sparsely firing neurons described above, the presence of neurons with such low probability of firing would likely exacerbate even further the limitation of spike sorting algorithms, thus highlighting the difficulty of detecting this type of neurons, in line with previous studies (Henze et al., 2000; Buzsaki, 2004; Shoham et al., 2006; Fujisawa et al., 2008). This limitation is important because sparsely firing neurons have been related to high level cognitive processes (Quian Quiroga et al., 2008; Quian Quiroga, 2012), processing of sensory inputs (Olshausen and Field, 2004) and have been found all over the neocortex (Kerr et al., 2005; Shoham et al., 2006). Moreover, sparsely firing neurons usually carry high amounts of information per spike (Quian Quiroga et al., 2007; Quian Quiroga and Panzeri, 2009) and their responses can be strikingly selective (Quian Quiroga et al., 2007), show invariance (Quian Quiroga et al., 2005) and multimodality (Quian Quiroga et al., 2009). Furthermore, it has been shown that the bursting of a single neuron can modify the global brain state (Li et al., 2009) or even the behavior of an animal (Brecht et al., 2004; Houweling and Brecht, 2008). Altogether, these results highlight the need for further improvements of the spike sorting methods presently available, paying special attention to their performance with sparsely firing neurons.

### 4.3 Tetrodes and stereotrodes

To improve the number of recorded units, a series of studies have proposed the transition from single electrode recordings to the use of tetrodes or stereotrodes (Gray et al., 1995; Harris et al., 2000; Blanche et al., 2005). These probes provide additional information by recording the firing of the neurons from different points (Harris et al., 2000) and they have been shown to provide better clustering outcomes (Gray et al., 1995; Harris et al., 2000). In our simulations we have only explored spike sorting limitations for single channel recordings. It is likely that with tetrodes the spike sorting algorithms would still identify a much lower number of neurons than the ones actually present in the recording, especially considering that with a tetrode it is possible to identify up to about 20 neurons in an area enclosing over 100 (Buzsaki, 2004). Interestingly, in a recording of cortical neurons *in vivo* using a 54 contact probe (Blanche et al., 2005), a comparison between sorting performances for a virtual tetrode (using only 4 adjacent contacts of the probe) and using a larger number of contacts showed that, in the case of the tetrode, 8 single units were identified, whereas when using the signal from all channels, 10 units were found: 7 corresponding to the ones found using the tetrode configuration and another 3 that were considered only one in the previous case. Interestingly, this particular error with the tetrode data – merging different single units into a same cluster – resembles the error described in Fig. 3 of our study.

The spike sorting process is determined by the shape and variance of the spikes, particularly the ones of neurons with the highest firing rates (contributing most of the spikes in a recording). This is related to the feature extraction phase of the algorithms, when the spikes are characterized in a feature space for posterior classification. The selected features reflect the main differences, which are determined by the large bulk of spikes of the high-firing neurons, and in turn, the features representing the spikes of the low-firing neurons tend to be underrepresented. In general, and beyond the particular issue of spike sorting, the clustering of groups with very different sizes is a known clustering problem (Ott et al., 2005).

The development of new recording probes that, for example, improve the quality of the signal transference from the tissue to the electrode and reduce the noise present in the signal (Keefer et al., 2008), opens the opportunity to dramatically increase the number of identified neurons from extracellular recordings. These developments should, however, be matched by advances in the methods used to process the recorded data. In this respect, the results presented here uncover the necessity of further improvement of spike sorting algorithms, particularly for the detection of sparsely firing neurons, as they are easily masked by neurons.

## Figures and Tables

**Table 1:** Correct detections (Hits). Minimum, maximum and average number of hits for the 3 experts. The left column indexes the results by the number of units present in the simulation. Performances for the 3 experts were very similar. The last three columns display the global statistics, averaging across the 3 subjects.

Neurons	Operator 1			Operator 2			Operator 3			Total		
	Mean	Min	Max	Mean	Min	Max	Mean	Min	Max	Mean	Min	Max
2	1.8	1	2	1.8	1	2	1.8	1	2	1.8	1	2
3	2.8	2	3	2.8	2	3	2.8	2	3	2.8	2	3
4	3.8	3	4	3.6	3	4	3.6	3	4	3.67	3	4
5	4.2	3	5	3.8	2	5	4.4	3	5	4.13	2	5
6	5.2	4	6	5.2	5	6	5.2	5	6	5.2	4	6
7	5	3	7	4.6	2	6	4.8	2	7	4.8	2	7
8	6.8	6	7	6.6	6	7	6.2	6	7	6.53	6	7
9	7.2	5	8	6.8	5	8	7	4	9	7	4	9
10	6.4	3	9	6.2	4	9	6.6	5	8	6.4	3	9
11	8	6	9	7.8	6	10	7.6	6	9	7.8	6	10
12	8.4	7	11	7.2	6	9	7.2	6	9	7.6	6	11
13	8.6	5	11	8.2	3	11	7	3	9	7.93	3	11
14	7	5	11	7.2	4	11	6.6	4	11	6.93	4	11
15	8.2	6	11	7.8	5	10	7.8	5	10	7.93	5	11
16	10.6	10	11	9.6	8	11	10.8	9	12	10.33	8	12
17	7.4	5	9	5.8	5	7	6.4	4	8	6.53	4	9
18	9.2	6	12	9.2	6	13	7.4	4	12	8.6	4	13
19	8.4	6	11	8.2	5	11	6.4	5	8	7.66	5	11
20	9.4	7	12	7.8	5	11	7.8	5	11	8.33	5	12

**Table 2:** False positives. Minimum, maximum and average false positives for the 3 experts. The left column indexes the results by the number of units present in the simulation. As in Table 1 results for different experts were very similar. The last three columns display the global statistics, averaging across the 3 subjects.

Neurons	Operator 1			Operator 2			Operator 3			Total		
	Mean	Min	Max	Mean	Min	Max	Mean	Min	Max	Mean	Min	Max
2	0.2	0	1	0	0	0	0.2	0	1	0.13	0	1
3	0	0	0	0	0	0	0.2	0	1	0.07	0	1
4	0	0	0	0	0	0	0	0	0	0	0	0
5	0.4	0	1	0.4	0	1	0.2	0	1	0.33	0	1
6	0	0	0	0	0	0	0.2	0	1	0.07	0	1
7	0.6	0	2	0.6	0	2	0.6	0	2	0.6	0	2
8	0.4	0	1	0.2	0	1	0.6	0	1	0.4	0	1
9	0.4	0	1	0.6	0	1	0.8	0	1	0.6	0	1
10	0.6	0	1	0.4	0	1	0.4	0	1	0.47	0	1
11	0.4	0	2	0.2	0	1	0.2	0	1	0.27	0	1
12	0.8	0	1	1.2	1	2	1.4	1	2	1.13	0	2
13	0.8	0	2	1	0	3	1.2	0	3	1	0	3
14	1.8	1	3	1.8	0	3	2.4	0	4	2	0	4
15	1.6	0	3	1.4	0	2	1.2	0	2	1.4	0	3
16	0.8	0	1	1.2	1	2	0.4	0	1	0.8	0	2
17	2.6	2	4	3.4	2	4	2.8	2	4	2.93	2	4
18	2.4	2	3	1.6	0	2	2.2	2	3	2.07	0	3
19	2.4	1	3	2	1	4	2.8	2	4	2.4	1	4
20	2.2	1	3	2.6	1	4	2.8	2	4	2.53	1	4
