# Supplementary material for: How many neurons can we see with current spike sorting algorithms?
Source: J Neurosci Methods. 2012 Oct 15;211(1):58–65. doi: 10.1016/j.jneumeth.2012.07.010 (PMC3657693; doi:10.1016/j.jneumeth.2012.07.010)
Supplement: Supplementary file 1 [file mmc1.doc]

### Figure S1


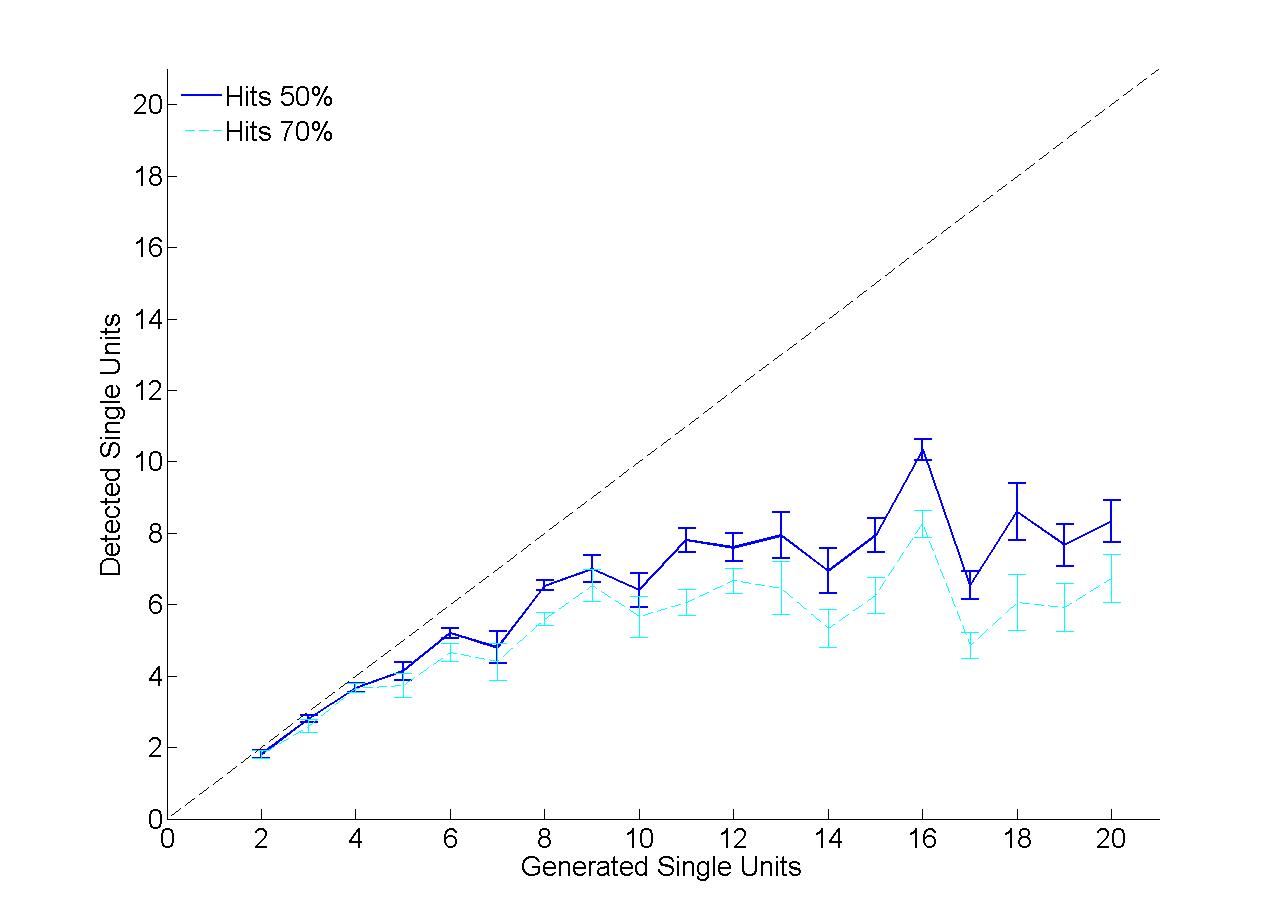
**Figure S1: Averaged hit performance for different values of percentage requirement**. When the number of spikes in the simulations was low, performances were similar for both criteria, but they differed as the number of neurons in the simulation increased. The presence of an asymptotic value for detection was common for both values.

### Figure S2


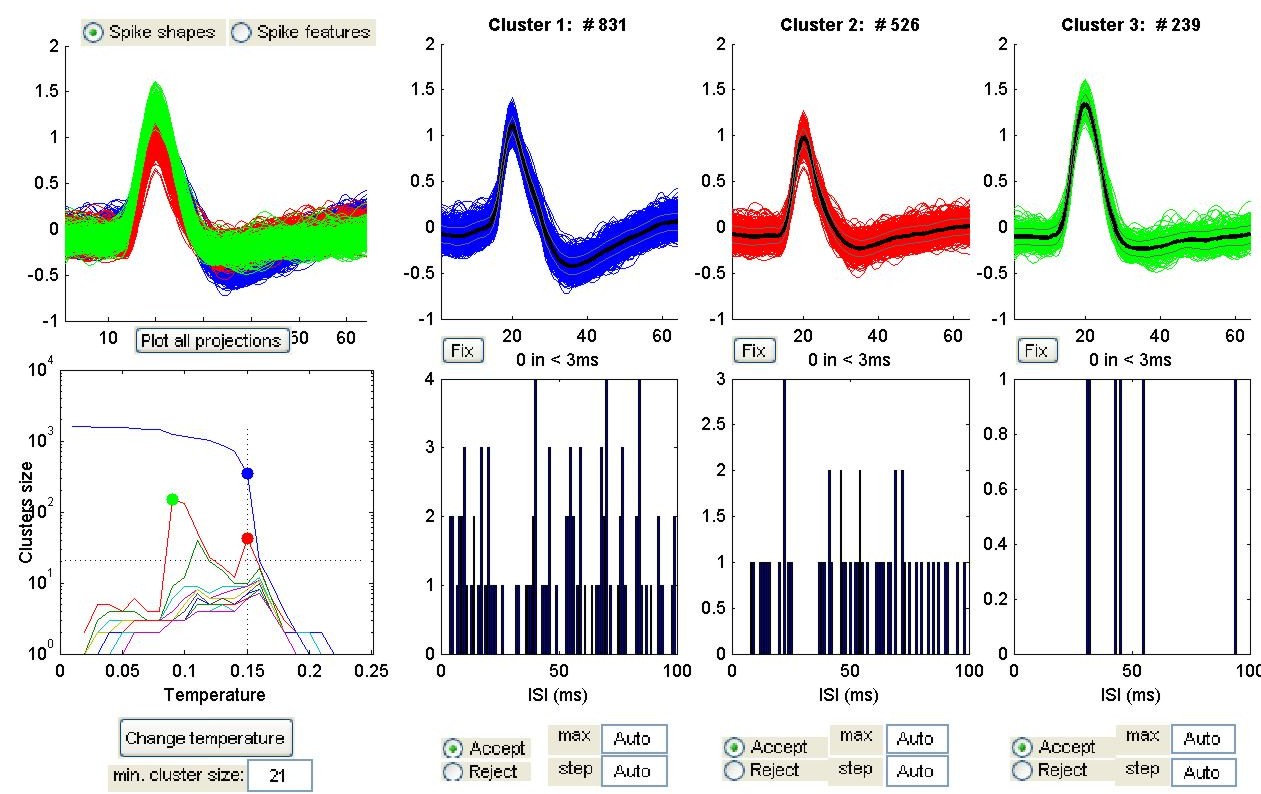
**Figure S2: Sorting of the spikes identified as cluster 1 in Figure 3.** When sorting the spikes on their own (no multiunit or other single units included in the spikes to be sorted) the algorithm reached the optimal solution, extracting the three units present in the cluster with great accuracy.
